# Review of the Potential of the Ni/Cu Plating Technique for Crystalline Silicon Solar Cells

**DOI:** 10.3390/ma7021318

**Published:** 2014-02-18

**Authors:** Atteq ur Rehman, Soo Hong Lee

**Affiliations:** Green Strategic Energy Research Institute, Department of Electronics Engineering, Sejong University, 98 Gunja-dong, Gwangjin-gu, Seoul 143-747, Korea; E-Mail: atteq@sju.ac.kr

**Keywords:** metallization, solar cell, efficiency, screen printing, photovoltaic, contact resistance, nickel/copper, metal plating, light induced plating (LIP), adhesion

## Abstract

Developing a better method for the metallization of silicon solar cells is integral part of realizing superior efficiency. Currently, contact realization using screen printing is the leading technology in the silicon based photovoltaic industry, as it is simple and fast. However, the problem with metallization of this kind is that it has a lower aspect ratio and higher contact resistance, which limits solar cell efficiency. The mounting cost of silver pastes and decreasing silicon wafer thicknesses encourages silicon solar cell manufacturers to develop fresh metallization techniques involving a lower quantity of silver usage and not relying pressing process of screen printing. In recent times nickel/copper (Ni/Cu) based metal plating has emerged as a metallization method that may solve these issues. This paper offers a detailed review and understanding of a Ni/Cu based plating technique for silicon solar cells. The formation of a Ni seed layer by adopting various deposition techniques and a Cu conducting layer using a light induced plating (LIP) process are appraised. Unlike screen-printed metallization, a step involving patterning is crucial for opening the masking layer. Consequently, experimental procedures involving patterning methods are also explicated. Lastly, the issues of adhesion, back ground plating, process complexity and reliability for industrial applications are also addressed.

## 1. Introduction

Developing an improved method for the metallization of silicon solar cells is essential for realizing greater efficiency. Currently, contact formation using screen printing is the dominant technology in the silicon based photovoltaic industry, as it is simple and fast. However, metallization of this kind has the disadvantage in having a lower aspect ratio and higher contact resistance, limiting solar cell efficiency. The mounting cost of silver pastes and decreasing silicon wafer thicknesses encourages silicon solar cell manufacturers to develop fresh metallization techniques that involve a lower quantity of silver usage with no need to rely on the pressing process of screen printing. The photovoltaic industry also needs for solar cells to have superior performance with a lower usage of silver pastes, as has been set out by the international technology roadmap for photovoltaics (ITRPV) [1]. In this regard, a great deal of investigation has already been conducted on different metallization techniques as an alternative to screen printing technology. The growth of metallization techniques with minor production costs and higher efficiencies is critical for the progression of solar cell manufacturing technology. These facts has been realized and a workshop dedicated for the metallization of crystalline silicon solar cells has been organized for the first time in Utrecht, The Netherlands, in 2008 [2]. After the first successful workshop, the series [3,4] was continued, and the fourth metallization workshop was held more recently in Constance, Germany, in May 2013 [5]. All of these workshops have helped improve understanding of the current state and recent advances of metallization technology.

Among the promising metallization techniques, the formation of metal contacts using Ni/Cu plating techniques is assumed to be one of the most viable candidates for future use as contact technology for the manufacture of solar cells. Using Ni/Cu plating techniques is a good solution to improve the efficiency of metallization processes with precision and low contact resistance. The technique can be realized at lower material costs, and is suitable for use in mass production. The standard processing sequence of Ni/Cu contact formation involves two steps: in the first step, (i) Ni metal film is plated on to the solar cell surface, followed by (ii) the final step of plating a copper metal film on top of the Ni. This Cu film over the Ni film serves as a conduction layer/contact for the solar cell [6–10]. The formation of metal contacts using electroplating processes is a well-developed technique that can only be used for conductors. As the electroplating process cannot be realized on semiconductors, an auto-catalytic method of Ni electroless plating (used to deposit nickel phosphorus or nickel boron) has to be adopted in order to make contacts for the semiconducting surface of the cell. This Ni electroless plating method has already been assessed and broadly employed in the formation of contacts for silicon solar cells [11–13]. The use of Ni as a seed layer makes it possible for metals to be plated on semiconducting or non-conducting materials. The process of Ni electroless plating requires a complexing agent, a buffer agent and a reducing agent that reacts with metal ions to form metal film. Apart from the conventional electroless plating of Ni seed layer, it is plated in the presence of illumination, referred to as light induced electroless plating (LIEP) [14,15].

**Figure 1.**
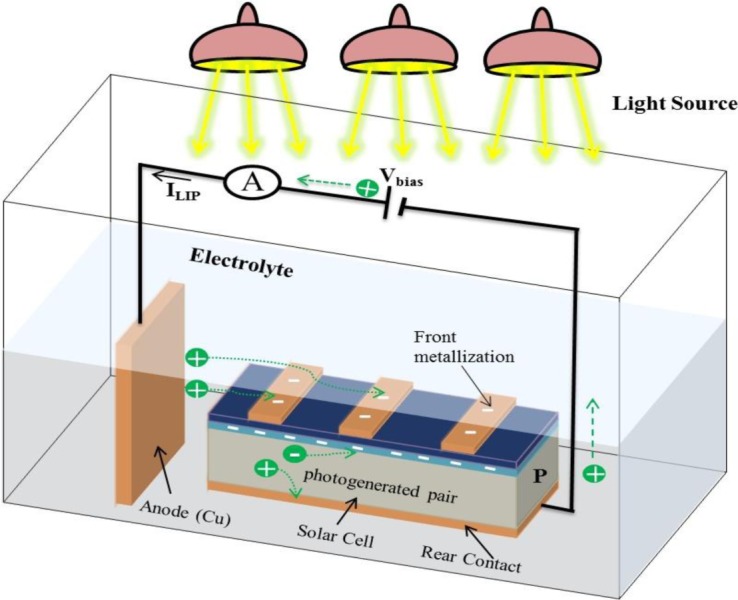
Schematic diagram of the light induced plating (LIP) based Cu electroplating process [16].

The approach of light induced plating (LIP) [16,17] developed by Fraunhofer ISE, is adopted and widely used for plating the silicon solar cell by many research groups. In the process of the LIP technique, a sample composed of n/p junction and an aluminum back contact formed by screen printing is immersed in a plating bath. The sample immersed in the plating solution is applied with a protective potential, so that rear of the cell can be made more cathodic, which results in the lowering of the aluminum corrosion. The protective potential not only prevents the erosion of aluminum but also helps to operate the cell closer to short circuit conditions and hence increases the plating uniformity [18]. The applied bias increases the operating current density of the cell, resulting in faster plating rates. The LIP arrangement is useful in controlling the height of the plated metal finger by varying the intensity of light or process velocity speed. Moreover, the LIP is considered to be favorable for the metallization of solar cells, as the metallization for the front grid as well as the fully aluminum doped rear side can be achieved at the same time. The schematic of such Cu based LIP plating process is shown in Figure 1.

In this paper a detailed overview of the metallization technology based on the Ni/Cu plating process will be given. The experimental procedures involved for Ni seed layer formation by various methods and LIP Cu plating will be discussed. Moreover, the current and future prospects of such low cost metallization technology for crystalline silicon solar cells will be addressed.

## 2. Need for an Alternate Metallization Scheme

The contact formation by the screen printing technique is most widely used for the crystalline silicon solar cells production. The key feature is higher through put with simple processing steps of co-firing the front and rear of the cells. Photovoltaic modules with efficiencies looming 19% can be realized for mono type crystalline silicon solar cells. However, there are certain factors involved with screen printed contacts that tend to limit solar cell performance. Factors such as fill factor and finger widths are the major ones. The fill factor for screen printed cells typically lies in the range of 75%–78%, which is because of the high contact resistance, lower metal conductivity and junction shunting presented by screen printed contacts. On the other hand, a fill factor of 81%–82% can be achieved with metallization based on a photolithographic process [19,20]. The finger widths of screen printed metallized solar cells are typically ≥100 μm, which contributes to an increase in shading loss. Shading losses can be decreased by lowering the widths with a double print (print-on-print) technique [21–23]. The print-on-print technique can increase cell efficiency by lowering the widths; however, the alignment issues still limit the high throughput production on an industrial scale.

**Figure 2.**
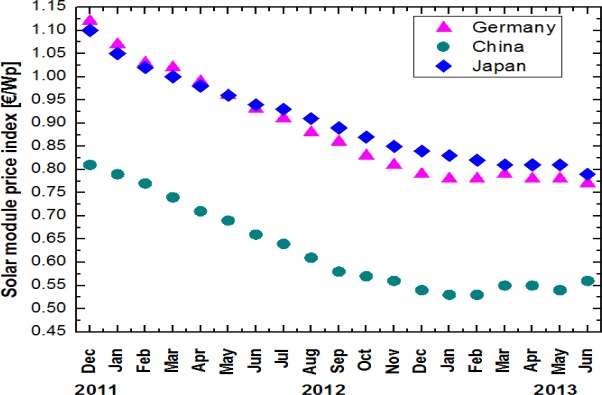
Development in the photovoltaic module prices in China, Germany and Japan.

The cost of metallization plays a vital role and is one of the most important economic factors in today’s industrial photovoltaic production. A report from Fraunhofer ISE indicates that up to 40% of cell processing costs is consumed by using expensive Ag paste [24]. An overall decrease in the price index for the solar module has occurred globally. Figure 2 shows the module price evolution in Germany, China, and Japan from January 2011 to June 2013 [25]. The photovoltaic business therefore requires an alternate scheme of metallization, as the mounting price of Ag affects it adversely.

**Figure 3.**
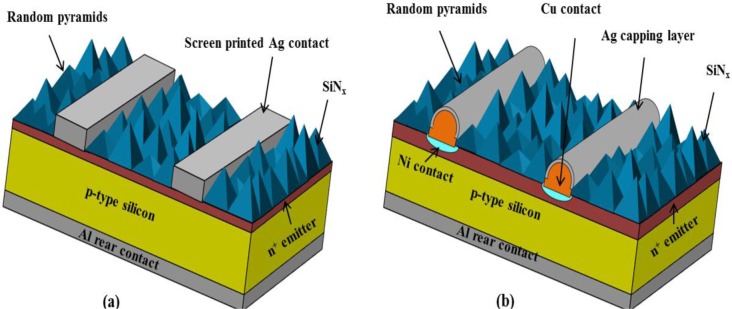
Solar cell structures composed of metallization scheme based on (**a**) Screen printed Ag paste; and (**b**) Ni/Cu/Ag plating.

The limitations of screen printed contacts in respect to lower fill factors, higher shading losses and the use of expensive Ag pastes specifically displays the need for an alternate metallization scheme. In order to produce high performance solar cells, several efforts including selective emitters, buried contacts cell structure, inter-digitated back contacts (IBC) and passivated emitter rear locally diffused (PERL) structure have already been tried [26,27]. A higher efficiency of 25% has already been achieved with the PERL structure [28]. However, the development of such a structure is expensive and too complex for commercialization. A Ni/Cu based metallization scheme has emerged as a future candidate for crystalline based silicon solar cells. The contacts formed by using such Ni/Cu based metallization can ensure improved results in terms of fill factors and efficiencies in comparison to conventional screen printed cells. The Ni/Cu metallization for silicon solar cells may not only produce a high performance solar cell but can also reduce the processing costs. By looking upon the incentives of relatively cheaper but almost equal conductive copper, companies and many research institutes have already started to adopt it as an alternate metallization scheme. Copper has a conductivity almost equal to Ag paste but is cheaper (nearly 100 times) than Ag (Table 1). Moreover, the Ni/Cu altogether offers ~2.5 times higher electrical conductivity and lower contact resistance at lower doping concentrations (Ns) in comparison to the conductivity offered by Ag paste for silicon solar cells [29]. Apart from the cost and conductivity, finer line widths can be made because of the self-aligned plating process used in the Cu metallization scheme. The contacts are usually formed with processing in a relatively lower temperature range (250–400 °C), which is considerably lower than the temperatures (750–850 °C) needed for Ag pastes. The use of a lower temperature process during the Cu based metallization scheme is useful for passivation layers as higher temperature certainly degrades the passivation schemes as well as can melt Al rear contact. The use of such low cost materials for metallization can certainly lower the euro/*W*_p_ price for solar cell modules. Copper, however, creates highly active recombination centers in silicon [30]; therefore, an effective barrier is required to prevent its diffusion in silicon. The Ni can form a promising barrier in between Cu and silicon, and it can also be useful in reducing contact resistance. A detailed comparison in terms of advantages and challenges for both metallization schemes is described in Table 2. The schematics in Figure 3 show the solar cell composed of metallization scheme based on screen printed Ag pastes and Ni/Cu/Ag plating technique. The next portion of the paper explains the processes involved in realizing such metallization scheme for silicon solar cells.

## 3. Ni/Cu Plating Technique

The increase of attraction for Cu-based metallization for ultra large scale integrated circuits (ULSI) is due to its greater resistance against electron migration and lower electrical resistance. However, Cu as a contacting material has some major weaknesses for application in metallization schemes. The Cu atoms can travel effortlessly through the SiO_2_ layer to the Si substrate, and being a deep level impurity in Si, it can disturb the electrical characteristics of the cell. The traps generated by these impurities act as generation and recombination centers, decreasing the minority carrier life-time in substrates [33,34]. To prevent the Cu from being diffused in the Si, a diffusion barrier is required. Ni as a seed layer can promote adhesion between Cu and Si and it can act as a diffusion barrier to the migration of Cu atoms in to Si substrates [35]. The two step metallization concept (seed-layer + LIP concept) [36] is suitable for obtaining higher efficiencies. In the case of the Ni/Cu plating technique, Ni can serve as a seed layer, while Cu as a main conducting layer can be deposited by electroplating technique. Thus the metallization technique, when using such an approach, involves two main steps:

- Ni seed layer formation
- Cu electroplating technique

**Figure 4.**
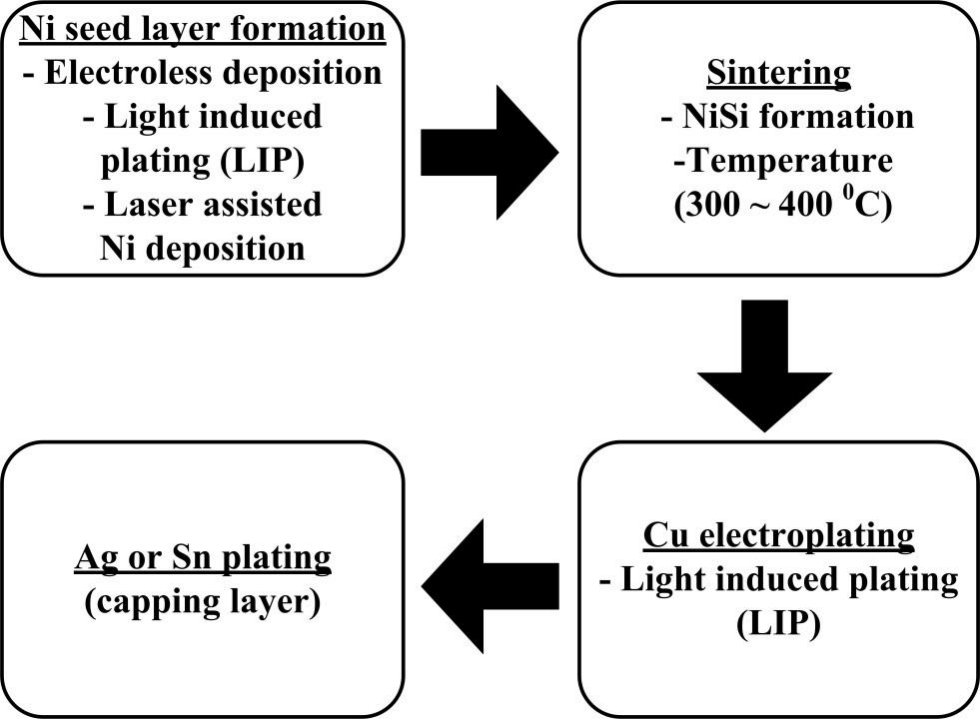
Steps involved in formation of the metallization scheme based on Ni/Cu/Sn or Ag metallization scheme.

After depositing the Ni/Cu stacks, a thin capping layer of tin (Sn) or silver (Ag) is generally deposited. This capping stack above Cu serves to protect these Cu conducting lines from being oxidized and to facilitate the soldering of interconnecting tabs. An additional significant aspect of the capping layer is that it avoids the interaction between the Cu and EVA encapsulant. The steps involved to form the metallization scheme composed of Ni/Cu/Sn or Ag stacks are shown in Figure 4.

### 3.1. Ni Seed Layer Formation

In the first step, Ni as a metal seed layer can be formed by either method such as electroless plating technique, light induced plating or laser assisted deposition. Besides Ni, other transition metals such as titanium (Ti) or tungsten (W) can also be used as a seed layer. Nickel is the most suitable candidate since it works well as a diffusion barrier to Cu as well as providing the low contact resistance to doped silicon. The Ni electroless deposition along with low temperature anneal provided an opportunity to form low resistivity ohmic contact layers [37,38]. There are a number of ways to perform the electroless plating process by adopting the concept of the oxidation reduction reaction. The plating rates can be varied significantly by using a light assisted plating (LIP) technique [39]. Another very effective way to deposit the Ni seed layer is by using the laser assisted Ni deposition technique [40]. The method is very much advantageous as the anti-reflection coating (ARC), ablation step can be performed along with the formation of the Ni seed layer. The adequate thickness and evenness over the entire front side patterned grid are the basic requirements for depositing an effective Ni barrier layer. The prevention of copper from being diffused for a desired lifetime defines the effectiveness of the barrier layer. However, a thinner Ni layer is favored as the Ni conductivity is low in comparison to Cu.

#### 3.1.1. Electroless Plating

The Ni deposition process using an electroless plating technique is a well-established and suitable process. It is an autocatalytic process that does not rely on the application of electrodes potential to sample. The reaction taking place during the deposition process involves the reduction of metals cations by means of electrons supplied by the reducing agent. The plating bath for Ni electroless plating is composed of the following bath compositions, including reducing agent of sodium hypophosphite [41]:

- Nickel chloride, (NiCl_2_·6H_2_O) or nickel sulfate, (NiSO_4_[H_2_O]_6_) as a main source of Ni
- Sodium hypophosphite, (NaH_2_PO_2_·H_2_O) as a reducing agent
- Triammonium citrate [(NH_4_)_3_C_6_H_5_O_7_] as a buffer and mild complex agent for Ni

The addition of ammonium hydroxide (NH_4_OH) is also required in order to maintain the PH value in between 8 and 10 [6,11]. This plating process is based on a catalytic oxidation-reduction reaction between hypophosphite ions and Ni. The chemical reaction can be described as the sum of two simultaneous steps. The steps are outlined as follows:

$$\text{Step }(1):H_{2}{\mathrm{PO}}_{2}^{-}+H_{2}O\to {\mathrm{HPO}}_{3}^{2-}+2H^{+}+H^{-}$$ (1)

$$\text{Step }\left(2\right):2H^{-}+{\mathrm{Ni}}^{2+}\to \mathrm{Ni}+H_{2}$$ (2)

$$\text{Sum}:2H_{2}{\mathrm{PO}}_{2}^{-}+2H_{2}O+{\mathrm{Ni}}^{2+}\to \mathrm{Ni}+H_{2}+4H^{+}+2{\mathrm{HPO}}_{3}^{2-}$$ (3)

#### 3.1.2. Light Induced Plating (LIP)

Light induced plating (LIP) baths were used for the formation of Ni as a seed layer. The chemical reactions (catalytic oxidation-reduction) taking place during the LIP Ni deposition process are the same as for the electroless plating bath outlined in the previous section. However, the inclusion of a light source helps to adjust the electrochemical potential of the front and rear of the cell and increases the plating rates [42]. The photo voltage by the PN junction and the electronegativity of the substrates accommodate to govern the electron migration at the surface. Furthermore, the diffusion of these photo-generated electrons at the surface helps in the reduction of Ni^2+^ ions and contributes in the form of higher plating rates [12]. The chemical baths can be operated at relatively lower temperatures with the addition of a light source in comparison to conventional electroless plating (non-LIP). Yu-Han *et al.*, demonstrate light induced nickel plating (LINP) for p-type substrates having uniform metal surfaces and high intrinsic quality [14]. Apart, from higher plating rates with uniform metal surfaces, the process involves the difficulty associated with the process characterization, as well as the dependence of plating on cell performance [18].

#### 3.1.3. Laser Assisted Ni Deposition

The use of lasers for depositing Ni as a seed layer has the advantage of using a single step for opening the ARC as well as the seed layer formation. The process involves the immersion of the solar cell in to an electrolyte solution and the application of a laser to write the grid from above. The working principle of laser induced Ni deposition from an aqueous electrolyte solution depends on the temperature rise produced by the laser in the solution and the wafer [40]. Because of the heat produced at the wafer surface the ARC is ablated at the area exposed and the temperature rise in the electrolyte initiates decomposition of Ni particles. Moreover, at the same time, an electron hole pair generation occurs in the solar cell by the light induced. The generated electron moves to the top surface and favors the Ni deposition. A uniform quantity of metal throughout the process is required to deposit a worthy Ni layer by the laser induced process. The water containing Ni salts could be helpful for the homogeneous coating of the wafers. Although, the experience in laser induced deposition from liquids still needs to be developed, there has been some research in this area [43–45]. The ARC ablation step along with the Ni deposition process by means of the laser chemical metal deposition (LCMD) process can play a critical role in commercialization of Ni/Cu metallization for silicon solar cells. At Fraunhofer ISE, the LCMD process was applied for depositing Ni along with a nitride ablation step and followed by a Cu plating step [46]. Another laser based metal deposition process called laser transfer contact (LTC) was also used to deposit an Ni seed layer through an optically transparent glass and finger widths of less than 30 nm was achieved [47].

#### 3.1.4. Ni Silicide Formation

**Figure 5.**
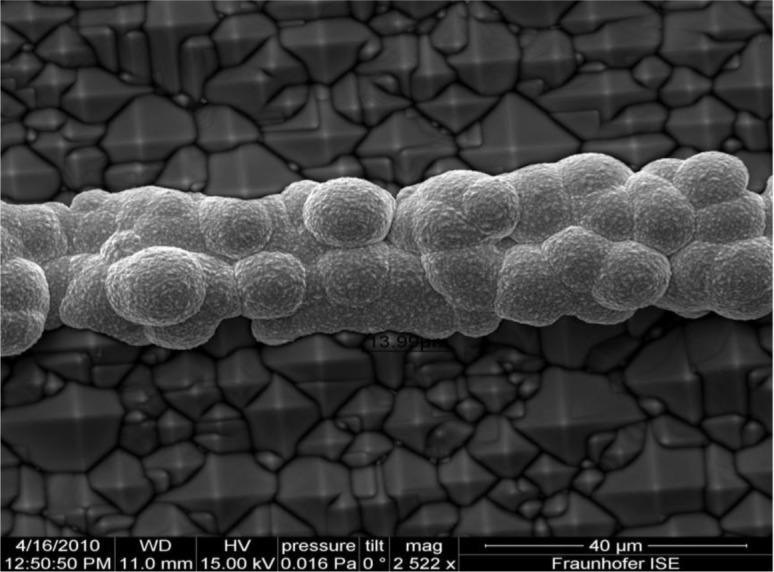
Plating of Ni by electrochemical deposition done by Mondon *et al.* at Fraunhofer ISE and presented at the 2nd metallization workshop for crystalline silicon solar cells, reprinted with permission from the authors (2014) [49].

A process known as a sintering process whereby heating in the ambiance of N_2_ gas is next to follow after Ni deposition which forms an alloy of Ni and silicon. The alloy formed acts as seed layers for Cu, which is usually prepared at higher temperatures (300–400 °C). This results in a reduction in metal semiconductor contact resistance [35]. The resistivity of Ni silicide is 14 μΩ.cm, which is comparable to Ti silicide (13–16 μΩ.cm) [6]. The Ni forms various phases with different compositions when heated at various temperature ranges, such as Ni_2_Si (200–300 °C), NiSi (300–700 °C) and NiSi_2_ (700–900 °C) [48]. The NiSi phase is identified as forming a lower resistivity state, and for that reason is beneficial for the metallization of silicon solar cells. The Ni plating process presented by Mondon *et al*. at 2nd workshop on metallization has been considered as a reference and is shown in Figure 5.

### 3.2. Cu Electroplating Process

Cu electroplating is usually done by the light induced plating (LIP) technique. The schematic in Figure 1 explains the working principle of the LIP process. The LIP technique involves the immersion of solar cell in an illuminated electroplating bath operated at room temperature. A Cu anode is placed in to the electrolyte bath, which is connected to the positive electrode of a dc voltage source. The sample (solar cell) is connected to the negative electrode of the battery. The electrolyte bath is composed of cupric sulfate, which helps in supplying copper ion (Cu^2+^), needed for the plating. The negative potential connected to the solar cell emitter helps the Cu ions to be attracted to the front metallized area. The photo-generated electrons in the samples recombine with the Cu ions and then the Cu is deposited on the existing metal stack (Ni seed layer). The main additives of the Cu plating bath are composed of cupric sulfate (CuSO_4_·5H_2_O) and sulfuric acid (H_2_SO_4_). The sulfuric acid plays a role of inducing the current flow at low voltage to increase conductivity [50]. The Cu deposition only occurs on the conductive surface, meaning not on the ARC covered areas but at the opened areas. The reactions taking place at each electrode during the Cu plating process are summarized in Table 3.

**Figure 6.**
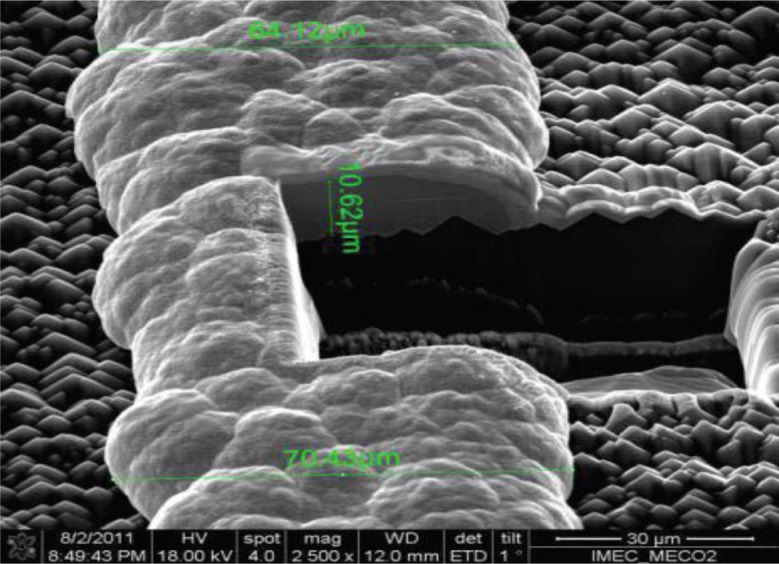
Scanning electron microscopy (SEM) images of the plated lines processed at MECO. Focus ion beam (FIB) cutting was used to analyze the cross-sectional images of the plated lines. The electroplated copper above nickel layer is covered by Ag capping layer at the top, reprinted with permission from the authors (2014) [52].

The working principle of LIP is same as the conventional plating process with the utilization of the photo-generation property of the solar cell. The LIP in comparison to conventional electro/electroless plating techniques has the advantages of having a uniform voltage distribution across the grid pattern (ensures a uniform current density) with stable baths (no reducing agents). Moreover, LIP is favorable particularly in the case of solar cell metallization, since it can perform the plating process for the front contact grids and can metallize the fully aluminum doped rear side of the cell. Higher aspect ratios and larger deposition rates (in the range of 0.1–2 μm/min) can also be achieved. These deposition rates can be controlled by observing the current (*I*_LIP_) in the external circuits as well as by regulating the light irradiance during the LIP process. An increase in the deposition rates can be achieved with higher *I*_LIP_ currents and larger light irradiance. However, at higher deposition rates the deposited material quality is poorer (higher porosity) as compared to the LIP process at lower rates [51]. Figure 6 shows SEM image for Ni/Cu/Ag stacks processed at MECO by Tous *et al* [52].

## 4. Experimental Section

**Figure 7.**
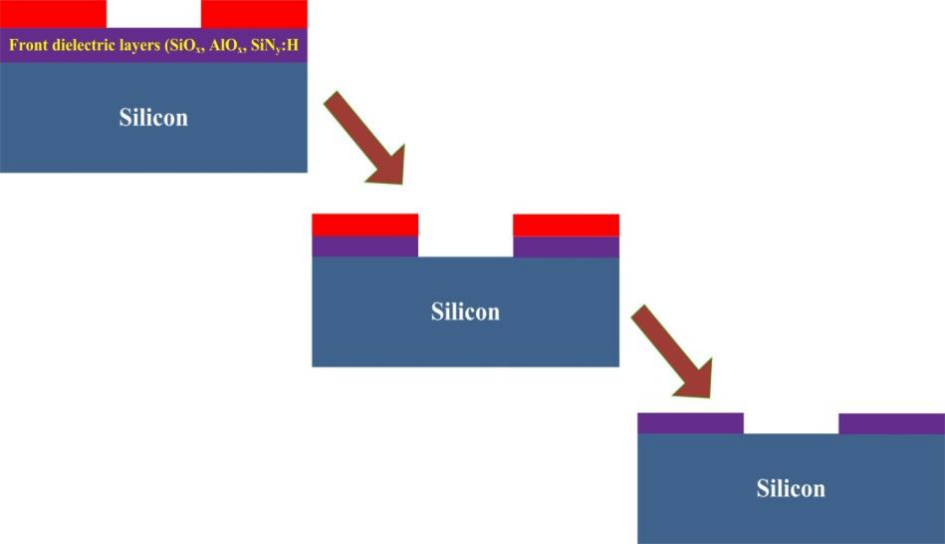
Schematic mask and etch sequence. Mask patterning is done initially to open ARC followed by a process of photoresist removal.

The sample composed of an n/p junction has to be submerged in the solutions containing the Ni electroless plating solution, followed by sintering process, to prepare a seed layer for Cu plating. Prior to performing the Ni electroless plating process, the ARC (anti reflection coating) has to be opened. There are a number of methods that can be used to open the ARC. One such method is the mask and etch sequence. Here the photoresist mask is patterned initially by using a photolithography process. The unprotected ARC is then etched away exposing the emitter area. The etching of ARC is usually done by the use of hydrofluoric acid followed by the striping of the mask layer by an organic solvent. The photolithography process which can be used for patterning the mask layer has the ability to pattern lines with lower widths. The schematics in Figure 7 show a mask and etch sequence that can be adapted for patterning the contacts for silicon solar cells.

**Figure 8.**
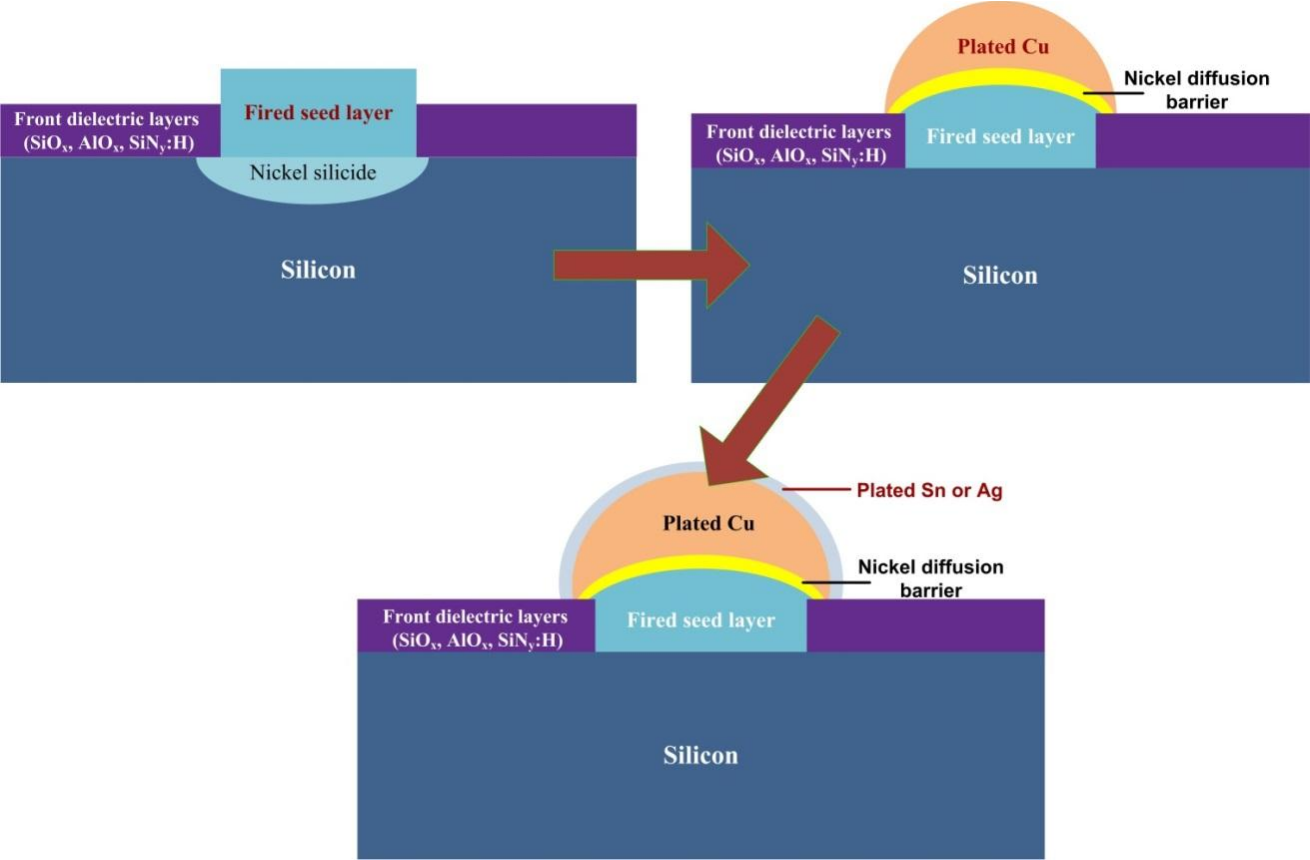
Schematic structures for the steps involved in the formation of metallization scheme based on Ni/Cu/Sn or Ag stacks.

**Figure 9.**
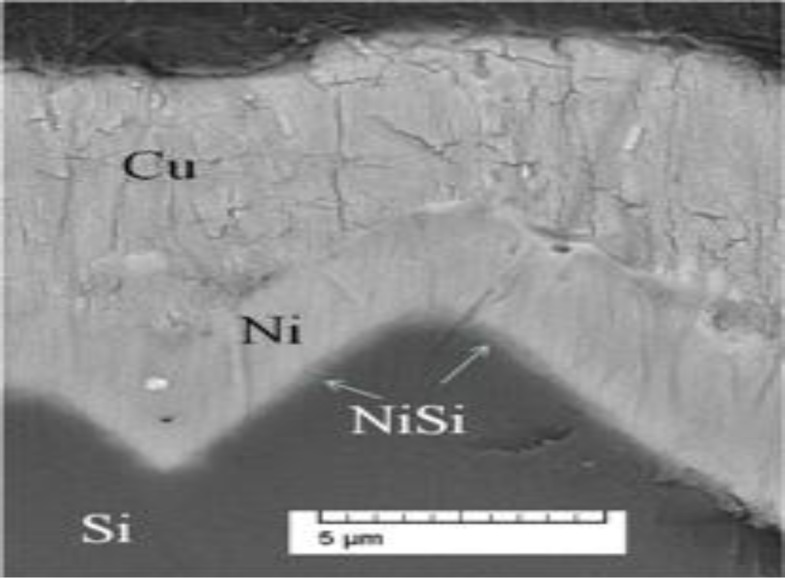
SEM cross sectional image of a mechanically polished NiSi/Ni/Cu stacks on textured silicon presented by Nguyen *et al.*, with permission from European Photovoltaic Solar Energy Conference (EUPVSEC), copyright and permission section (2013) [59].

After the ARC opening, Ni can be plated on exposed area for the formation of a seed layer. The electroless plating or LIP bath consisting of a Ni source in the form of metal salt (NiCl_2_ and NaH_2_PO_2_), performs the process of forming this Ni metal layer. The Ni metal begins to deposit on the silicon surface in the form of the Ni layer [53]. The Ni layer deposited needs to be sintered at lower temperature in order to form Ni silicide [54]. Min *et al*. worked on optimizing the nickel sintering process and were able to manufacture 18.15% efficient PESC (passivated emitter solar cell) cell with selective emitters [55]. Cu electroplating using the LIP technique is the next step to be performed after the seed layer. The schematics in Figure 8 show a contact structure that can be formed by plating Ni and Cu followed by Tin (Sn) or Ag a capping layer. The SEM image in Figure 9 shows the mechanically polished NiSi/Ni/Cu stacks formed on textured silicon surface presented by Nguyen *et al.* [56].

The photolithographic based ARC opening method has the ability to pattern the lines with lower widths. However, the number of processing steps involved in such a method can limit the high throughput desirable for commercialization. There are a number of methods other than the photolithography process that has been applied for opening the ARC. The methods include the use of an etching pastes [57–59], laser transferred Ni seed layers [60], ARC opening by mechanical scribing [9] and aerosol jet for etching [61,62]. The use of the laser chemical metal deposition (LCMD) process is considered to be the most suitable process for industrial application. The method can certainly make the process simpler, as the nitride ablation and seed layer formation can be performed in a single step [43,44]. N. Wehkamp *et al.* at Fraunhofer ISE have worked on such type of laser chemical metal deposition methods, and reported an efficiency of 17.9% by forming a 40 μm wide line for solar cell fabricated on p-type CZ silicon substrate [46].

## 5. Cu Plating Contributions

**Figure 10.**
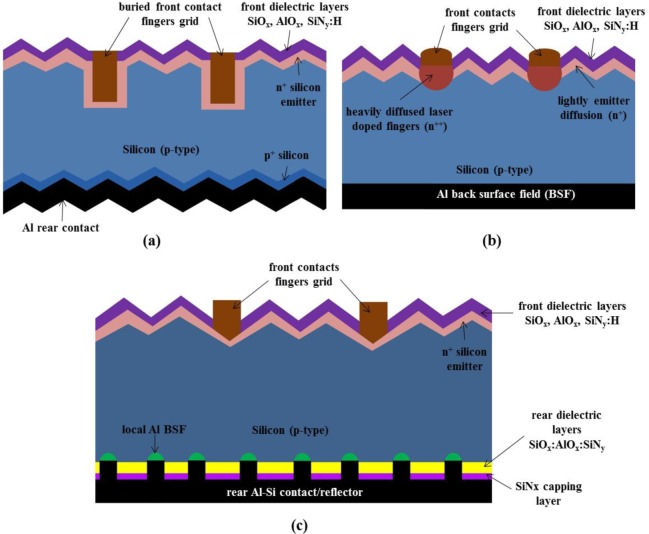
Solar cell structures most commonly adopted for Ni/Cu based metallization scheme. (**a**) Laser doped buried contact (LDBC) cell; (**b**) Laser doped selective emitter (LDSE) cell; (**c**) Passivated emitter rear cell (PERC).

In spite of the potential benefits of Ni/Cu contacts, its commercialization has been limited thus far. BP Solar has been alone producing laser grooved buried grid (LGBG) type cells between the years 1992 and 2008 [63]. The increased process complexity and convenience for the use of appropriate low cost production techniques are the primary reasons restricting its commercialization. The Ni/Cu as a front contact however, has been investigated by many research institutes including, Fraunhofer ISE [6–8,55,64–69]. The advancements in new cost effective patterning techniques and biased assisted light induced Ni/Cu plating have come up with solutions for complications and have helped in realizing practical low cost contacts that do not rely on Ag screen printing. In recent years, efficiencies of over 20% have been reported by various companies (Hyundai Heavy Industries, Kaneka, Schott Solar, Silevo, SunTech, TetraSun) and research institutes (Fraunhofer ISE, IMEC, Roth & Rau Research) with the cells based on Cu metallization. The investigation for cell structures, such as Heterojunction Cells, laser doped buried contacts (LDBG), laser doped selective emitter (LDSE) and passivated emitter and rear cell (PERC) type cells has been conducted for a metallization scheme based on the Cu plating technique. The process flow for LGBG type cells from BP Solar involves, laser grooving, Ni and Cu electroless plating followed by deposition of a thin Ag layer for solderability. Figure 10 shows cell structures of laser doped buried contact (LDBC), LDSE and PERC type solar cells that has been investigated for the Ni/Cu based metallization scheme.

**Figure 11.**
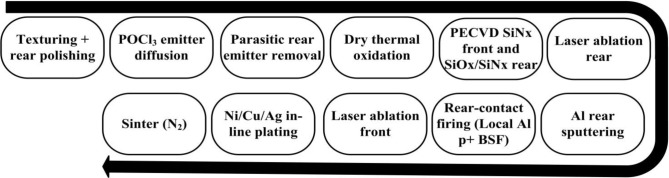
Process flow of PERC type solar cell processed at IMEC [73].

PERC type solar cell structures having efficiencies above 20% have already been demonstrated by several institutes with best cell efficiency of 21.4% at Fraunhofer ISE [70]. Schmid Group and Schott Solar demonstrated 20.9% efficient PERC type cell with the front side electroplated with Ni/Cu [71]. More recently, IMEC along with MECO presented i-PERC type solar cell [72]. The cells offer industry-applicable Ni/Cu plated front contacts with an average of 20.5% efficiency (>100 cells) and with the best cell efficiency of 20.79% [73]. IMEC emphasized the parameters like cell efficiency, module reliability and euro/*W*_p_ cost potential for i-PERC type cell by means of pilot-line processing tools. The research team was however successful in establishing of a simpler metallization sequence for Ni/Cu/Ag type contact having an advantage of minimal number of processing steps. The process flow of IMEC i-PERC type solar cell is shown in Figure 11.

The LDSE cell concept was pioneered at the University of New South Wales (UNSW), Australia. Hyundai Heavy Industries, in Korea have presented such LDSE solar cells using Ni/Cu based front side metallization having 19.8% efficiency [74]. UNSW and Shinsung Solar Energy have also presented LDSE solar cells with Ni/Cu based metallization with efficiencies of 19.3% and 19.6%, respectively [75–77]. Apart from LDSE and PERC type cells, Suntech Power has produced Pluto cells with stabilized efficiencies of 19% for Ni/Cu metallization [78]. Solar cell composed of Cu based metallization wrap through (MWT) cell structure was fabricated by Holger *et al.* at the University of Konstanz, Germany [7]. Kaneka and Roth & Rao Research adopted the Cu contacts for Heterojunction type cells and presented higher efficiencies of 23.5% and 22.3% respectively [79,80]. Some of the optimal results for solar cells composed of Cu plated front contacts are summarized in Table 4.

## 6. Future Challenges

Solar cells having superior performance at lower costs can be realized using the Ni/Cu plating approach. There has been good progress in various aspects of the process. Higher efficiencies (up to 21.4%), can be achieved with such low cost metallization schemes [70]. While taking into account the advantage of high efficiency potential over screen printed cells the cost of ownership of Ni/Cu plating needs to be considered. Although the economic advantage of Ni/Cu plated solar cells against their screen printed counterparts is striking. Yet, the plating process needs to be optimized entirely, in terms of cost and performance. If just the material price is considered, the saving potential is relatively high. However, consideration also has to be given to the additional process complexity and higher investment expenditure involved in replacing Ag printer/dryer with a setup comprising of laser, plating system and a sintering furnace. Nonetheless, in terms of material costs, Ni/Cu may be advantageous given the current higher price of Ag and the fact that demands for it is expected to increase in years to come. In a recent report from IMEC, a cost estimation for both of these metallization schemes was made which clearly favored the Ni/Cu plating scheme [73]. It is not only the cost of ownership but there are a number of issues to be resolved before a standard industrial process is presented. The major issues are (i) formation of a Ni seed layer uniformly, (ii) contact adhesion, (iii) back ground plating (iv) enhanced process complexity in comparison to Ag screen printing and more importantly the long term reliability.

### 6.1. Uniform Ni Seed Layer

The issue of depositing a uniform Ni layer is some extent solved by adopting processes such as electroless Ni plating [83] with a higher pH solution [84] and the light induced electroless plating method [14]. The use LIP for Ni plating bath may also be useful for faster plating rates and high throughput. Apart, from LIP the two staged (etching back unreacted Ni + replating step) is found to form a uniform Ni layer [85]. This method may not only form a uniform Ni layer but can also help to improve contact adhesion. A two-step Ni deposition process can also cover up the Si surface. For a complete covering of the Si surface an approach of depositing Ni seed layer with two steps Ni plating process, having a contact resistivity of 0.6 mΩ.cm^2^ has been demonstrated by Seran *et al.* at University of Konstanz, Germany [86].

### 6.2. Contact Adhesion

The problem of poor adhesion between metal and silicon is one of the main obstacles to the route of Cu based plating technology for solar cells. The justifications for low adherence have not been clarified completely. However, the use of seed layers, the environment for their realization and the progressive effects are well understood. The issue of improving the adhesion has been investigated by various research institutes [52,85,87]. The adhesion quality of the contacts formed can be assessed by performing various tests such as, a heat-quench test, a peel force test or pull strength tests. So far adhesions in the range 1–2.7 N/mm have been achieved for metallization based on Ni/Cu stacks [52]. Formation of an adhesive Ni barrier was also investigated at Fraunhofer ISE, where a two stage process for contact formation was reported [85]. The process contains steps of etching back of unreacted Ni followed by a re-plating process. The adoption of this two stage process resolved the problem of low adherence of Ni to Si with an excellent adhesion of up to 2.5 N/mm being achieved. The contact adhesion for Cu over Ni was also found to be influenced by the roles of bath ageing, acidity of the bath and cleanliness of the substrates. The acidic baths and an unclean surface affect the adhesion adversely while the stress present in films due to bath ageing has an impact on it as well [88,89].

### 6.3. Back Ground Plating

Another challenge is the prevention of back ground plating also known as ghost plating or parasitic plating. The existence of impurities, particles and cracks on the wafer surface is mainly responsible for this phenomenon [90]. This effect has been noted when a number of pinholes are featured at the passivation layer. The plating occurs at these locations and results in undesirable shading and aesthetical deprivation of the solar cells. The presence of such plating affects the cell performance adversely. This can cause reduction in *J*_sc_ due to shading of the front surface as well as junction shunting by the metal diffusion. Moreover, it may also lead to the increase in recombination velocity at the surface due to localized metal silicon contact. The primary causes of this unwanted phenomenon were demonstrated well by Barun *et al.*, [91]. The unwanted plating was found to be mainly of two forms:

- Point like effects
- Cracks

The inhomogeneities of SiN*_x_*:H layers or the presence of silicon residuals were found mainly responsible for the generation of point like effects. However, the cracks which can be formed by mechanical stress (ultrasonic treatment) or insufficient texturing may also leads to the occurrence of the back ground plating of this type. This phenomenon of undesired plating on the tiny spots in the middle of fingers could be resolved by appropriate cleaning prior to SiN*_x_*:H deposition. The piranha solution (H_2_O_2_ + H_2_SO_4_) was found to be very effective for such treatment. The handling of the wafers (low mechanical stress during processing) is also very critical to avoid the unwanted metallization on cell surface [91,92].

### 6.4. Process Complexity

The process complexity in comparison to the screen printed Ag metallization is also considered an issue for commercialization of Ni/Cu plating technique. In order to form a potential Cu electrode and an effective Ni seed layer with sintering process increases the number of steps. However, implementing the Ni/Cu technique for solar applications the reduction in the processing steps is highly demanded. A smarter approach could be combining a nitride ablation and a seed layer formation in a single step by using a laser process and a Ni electrolyte altogether [46]. Additionally, instead of electroless seed plating a faster and cheaper LIP technology can be adopted to create such contacts. The metallization process can be simplified further by applying the sintering process as a last step after full stack plating. At IMEC such thermal contact formation after Ni/Cu/Ag stacks formation were reported with promising results [93].

### 6.5. Long Term Reliability

One of the main issues restricting the commercialization of the Ni/Cu based metallization process is the long term reliability of Cu. As Cu is a deep level impurity in Si, it can have an impact on cell performance if it is being diffused in to Si. In order to determine the reliability of the solar modules, an IEC 61215 test has to be carried out. The requirements are ≤5% *p*_max_ loss after 200 thermal cycles (−40° to 85 °C) or 1000 h damp heat exposure (85 °C, 85% relative humidity). Research institutes such as Fraunhofer ISE and IMEC have conducted such reliability tests and these were satisfactory up to a point. At Fraunhofer ISE, the pseudo fill factor (PFF) as measure for contamination was monitored for 1700 h at 200 °C temperature and the electrical performance was observed to be unchanged [70]. Meanwhile at IMEC, the modules made with Ni/Cu metallization passed the reliability test (damp heat and thermal-cycling IEC 61215 extended tests), but this is still insufficient for long term reliability [73]. In another report, from Papet *et al.*, a damp heat test was conducted for Ni/Cu plated heterojunction cells showing only 3% of power degradation after 3000 h [80]. This shows the reliability of Ni/Cu plating on heterojunction cells with time and temperature. Although, there have been some encouraging results in terms of reliability for cells comprised of Ni/Cu contacts, complete testing is nevertheless required before industrial application.

## 7. Conclusions

The manufacturing costs of current silicon solar cells can be reduced by using inexpensive metals for metallization and thinner silicon substrates. This review has traced the prospective of Ni/Cu plating technique for metallization of silicon solar cells. The use of Ni for promoting the adhesion and as a barrier to Cu shows great promise with many new plating techniques. Cu has the potential to become a major contributor as a cost effective material for future metallization schemes for silicon solar cells. Promising results in terms of higher fill factors and open circuit voltage during the last few years has been achieved. The advancement in new patterning technique such as the use of aerosol jet, laser doping and laser based chemical metal deposition has also occurred with the progression of plating techniques. A combination of nitride ablation and a Ni seed layer formation can be useful for the reduction in process complexity.

Metallization based on Cu plating appears to be a good alternative to existing screen printing technology in terms of material costs and cell performance. However, a standard industrial process is still limited by several hurdles. The challenges of adhesion, back ground plating, and process complexity has been resolved to a certain extent, however long term reliability is considered to be the primary obstacle to the sustainable manufacturing technology. The strong focus on the formation of reliable Ni/Cu contacts can be inevitable for the prediction of ITRPV roadmap, of Cu being the future metal contact for silicon solar cells.

## Figures and Tables

**Table 1.** Assessment of various metals costs and properties [31,32].

Parameters	Ag	Cu	Ni
Cost [$/troy oz]	21.87	0.225	0.440
Conductivity [10^6^ S/m]	61.3	59.1	13.9
ρ [g/cm^3^]	10.49	8.92	0.50

**Table 2.** Conventional Screen printed cells *versus* cells based on Ni/Cu plating scheme.

Metallization scheme	Benefits	Challenges
Conventional Screen printed cells	Simple processSpeedy and mass production	Higher costRelatively low cell efficiencyHigh contact resistanceLow aspect ratio
Ni/Cu plated cells	Low costHigh aspect ratio (decrease in shading losses)Low contact resistanceHigher efficiencies	AdhesionBack ground platingProcess complexity

**Table 3.** Reactions taking place at anode (Cu), electrolyte solution (H_2_SO_4_/CuSO_4_) and cathode (substrate).

No.	Reaction places	Reaction
1)	Anode (Cu metal)	$\mathrm{Cu}+2e^{+}\to Cu^{2+}$
2)	Solution	$\begin{array}{l} \mathrm{Cu}SO_{4}\to Cu^{2+}SO_{4}^{2-}\\ H_{2}SO_{4}\to 2H^{+}+SO_{4}^{2-} \end{array}$
3)	Cathode (substrate)	${\mathrm{Cu}}^{2+}+2e^{-}\to \mathrm{Cu}$

**Table 4.** Optimal results for various high efficiency cell structures with front side metallized by the LIP based Cu plating technique.

R & D Centre	Type	Substrate	*V*_oc_ (mV)	*J*_sc_ (mA/cm^2^)	Fill Factor (%)	Efficiency (%)	References
Kaneka	Heterojunction	CZ	737.0	39.97	79.77	23.50	[79]
Roth & Rau Research	Heterojunction	–	734.0	38.1	79.9	22.3	[80]
Fraunhofer ISE	PERC	FZ	679.0	38.8	81.5	21.4	[70]
Schott Solar	PERC	CZ	665.0	39.9	80.5	21.3	[81]
IMEC	PERC	CZ	649.8	39.3	78.3	20.79	[73]
IMEC	Rear junction	CZ	676	38.4	79.2	20.5	[82]
Fraunhofer ISE	–	FZ	646.4	38.86	80.8	20.3	[64]
Hyundai Heavy Industries	LDSE	CZ	635.0	39.1	79.8	19.8	[74]
Shinsung Solar Energy	LDSE	CZ	644.0	38.75	78.72	19.64	[76]
IMEC	PERC	CZ	655.9	38.3	77.8	19.6	[52]
UNSW	LDSE	CZ	638.3	38.43	78.82	19.33	[75]

Notes: *V*_oc_: open circuit voltage; *J*_sc_: short circuit current density.
